# Alternative Therapeutic Options to Antibiotics for the Treatment of Urinary Tract Infections

**DOI:** 10.3389/fmicb.2020.01509

**Published:** 2020-07-03

**Authors:** Paul Loubet, Jérémy Ranfaing, Aurélien Dinh, Catherine Dunyach-Remy, Louis Bernard, Franck Bruyère, Jean-Philippe Lavigne, Albert Sotto

**Affiliations:** ^1^VBMI, INSERM U1047, Université de Montpellier, Service des Maladies Infectieuses et Tropicales, CHU Nîmes, Nîmes, France; ^2^VBMI, INSERM U1047, Université de Montpellier, Service de Microbiologie et Hygiène Hospitalière, CHU Nîmes, Nîmes, France; ^3^Service des Maladies Infectieuses, AP-HP Raymond-Poincaré, Garches, France; ^4^PRES Centre Val de Loire, Université François Rabelais de Tours, Tours, France; ^5^Service des Maladies Infectieuses, CHU Tours, Tours, France; ^6^Service d’Urologie, CHU Tours, Tours, France

**Keywords:** urinary tract infection, alternative therapeutics, vaccines, nutraceuticals, immunomodulating agents, probiotics, cranberry, bacteriophages

## Abstract

Urinary tract infections (UTIs) mainly caused by Uropathogenic *Escherichia coli* (UPEC), are common bacterial infections. Many individuals suffer from chronically recurring UTIs, sometimes requiring long-term prophylactic antibiotic regimens. The global emergence of multi-drug resistant uropathogens in the last decade underlines the need for alternative non-antibiotic therapeutic and preventative strategies against UTIs. The research on non-antibiotic therapeutic options in UTIs has focused on the following phases of the pathogenesis: colonization, adherence of pathogens to uroepithelial cell receptors and invasion. In this review, we discuss vaccines, small compounds, nutraceuticals, immunomodulating agents, probiotics and bacteriophages, highlighting the challenges each of these approaches face. Most of these treatments show interesting but only preliminary results. *Lactobacillu*s-containing products and cranberry products in conjunction with propolis have shown the most robust results to date and appear to be the most promising new alternative to currently used antibiotics. Larger efficacy clinical trials as well as studies on the interplay between non-antibiotic therapies, uropathogens and the host immune system are warranted.

## Introduction

Urinary Tract Infections (UTIs) are frequent bacterial infections (Silverman et al., 2013) especially in women, estimated to affect more than one in every two women at least once in her lifetime (Foxman, 2014; McLellan and Hunstad, 2016). Uropathogenic *Escherichia coli* (UPEC) is the main pathogen isolated from patients with UTIs (>85%) (Flores-Meireles et al., 2015), while other Gram-negative rods (e.g., *Proteus mirabilis*, *Klebsiella pneumoniae*) and Gram-positive cocci (e.g., *Staphylococcus saprophyticus*, *Enterococcus faecalis*) are responsible for the remaining cases (Flores-Meireles et al., 2015). UTI recurrence is defined by the occurrence of more than two episodes in 6 months, or three in 12 months (Professionals, 2019). The annual incidence of UTI in women is estimated to be 30 per 1000 subjects (Laupland et al., 2007), with approximately 20–40% experiencing recurrence within 6–12 months (Ikähelmo et al., 1996; Foxman, 2014). The epidemiology of UTI changes significantly in the healthcare environment. Urethral catheterization is strongly associated with UTI, and the risk of infection increases with the length of catheterization (Suetens et al., 2018). Catheter-associated UTI (CAUTI) is the most common nosocomial infection (Suetens et al., 2018). CAUTI affects both sexes, with long-term urinary catheterization of both men and women almost invariably leading to detection of bacteria in the urine (bacteriuria). Long-term catheterization carries a daily risk of 3–7% for the development of symptomatic CAUTI (Saint et al., 2009).

The urinary tract is normally sterile, with the exception of the flora of the distal urethra which is diverse and reflects both the digestive flora, the cutaneous flora and genital flora (*Lactobacilli* in women). There are several physiological mechanisms to prevent the host from the development of an ascending infection. First, the urethra itself, which is an obstacle to the intravesical inoculation; second, the physicochemical characteristics of normal urine (osmolarity, pH, organic acid content) that makes growth of most of the bacteria colonizing the urethra difficult; third urination that eliminates most of the bacterial population; fourth the presence in the urine of glycoproteins and oligosaccharides acting as soluble receptors to capture bacteria and enhance their clearance. Finally, in case of bacterial colonization, three factors contribute to avoid the invasion of the mucous membrane (Sobel, 1997): (i) the presence of inhibitors of bacterial adhesion to the surface of urothelial cells (Tamm-Horsfall protein, mucopolysaccharides); (ii) the existence of a local bactericidal effect (independent of inflammatory response or immune response); (iii) a process of exfoliation of the infected urothelial cells. The occurrence of UTI implies either a flaw in these defense mechanisms or the development in the urethral flora of a virulent bacteria, termed uropathogenic. Only a minority of *E. coli* strains, are endowed with uropathogenicity by the production of one or more adhesins (fimbriae): (i) type 1 allowing low urinary tract colonization, (ii) type P inducing pyelonephritis by modification of ureteral peristalsis in binding to glomerulus and endothelial cells of vessel walls helping *E. coli* to cross the epithelial barrier to enter the bloodstream and causing hemagglutination of erythrocytes and by decreasing the renal filtrate flow due to the formation of dense bacterial communities within the tubular lumen (Roberts, 1991; Melican et al., 2011), and (iii) non-fimbrial adhesins such as UpaB that facilitate *E. coli* adherence to extracellular matrix proteins and colonization of the urinary tract (Paxman et al., 2019). An increased adherence of *E. coli* to uroepithelial cells is observed in patients with recurrent UTIs compared to healthy controls (Schaeffer et al., 1981). Moreover, it has been demonstrated that UPEC can invade and replicate within the bladder cells to form intracellular bacterial communities (Mulvey et al., 2001), which can be frequently found in urothelial cells in women with symptomatic UTIs (Rosen et al., 2007) and may act as a source of recurrence in women with same-strain recurrent UTIs (Beerepoot et al., 2012a). Finally, biofilm formation is a critical aspect of CAUTI (Soto et al., 2006; Beerepoot et al., 2012a). Mechanisms of recurrence in UTIs are not fully characterized. Besides pathogen virulence factors, an impaired mucosal immune response (with urinary IgA involved in the UPEC clearance from the bladder mucosa) of the urogenital tract may have a role in the host-pathogen process (Ingersoll and Albert, 2013; Abraham and Miao, 2015).

Long-term low dose antibiotic use is currently the keystone of the preventive treatment for UTI recurrence. Indeed, prophylactic antibiotics have been shown to decrease UTI recurrence by 85% compared to patients with placebo (relative risk (RR) 0.15, 95% confidence interval (95%CI) 0.08 to 0.28) (Albert et al., 2004). Moreover, with regard to urinary tract conditions such as neurogenic bladder, it has been suggested that weekly cycling of antibiotics could be the most optimal preventative strategy (Salomon et al., 2006; Dinh et al., 2019). Indeed, this original strategy seems effective with only a limited ecological effect on native gut microbiota according to long-term follow-up (Poirier et al., 2015). However, prolonged antibiotic use often results in the emergence of multidrug-resistant organisms (Beerepoot et al., 2012b) and increases the cost of care. Consequently, the development of new therapeutic options to prevent and treat UTIs, and most particularly recurrent UTIs, are of interest.

**FIGURE 1:**
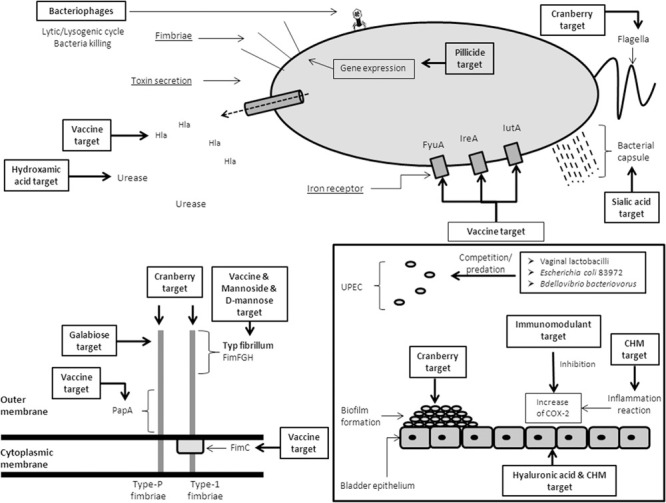
Recapitulative scheme of the alternative therapeutic options against UPEC. CHM: Chinese Herbal Medicine.

This review aims to describe all the existing non-antibiotic treatment options in UTI (Table 1 and Figure 1).

**TABLE 1:** Non-antibiotic therapeutic options for the treatment of urinary tract infections.

Therapeutic options	References	Mechanism	Benefits	Drawbacks
**Vaccine**				
Targeting adhesion	(O’Hanley et al., 1985; De Ree and Van den Bosch, 1987; Riegman et al., 1988; Wizemann et al., 1999; Langermann et al., 2000; Roberts et al., 2004; Poggio et al., 2006; Habibi et al., 2016)	• Block the liaison adhesin-host cell receptor (pili vaccine) • Reduction of adhesion and protection against cystitis (FimH vaccine)	• Decrease the bacterial colonization • Protection of the bladder and the kidneys	• Heterogeneity of the proteins of the bacterial membrane
Targeting capsule	(Kaijser et al., 1983; Roberts et al., 1993; Kumar et al., 2005; Stenutz et al., 2006)		• Promising animal model results	• No human studies • Great heterogeneity in antigen used making creation of a vaccine with broad protection difficult
Targeting toxins	(O’Hanley et al., 1991; Ellis and Kuehn, 2010)	• Reduction of renal injury	• Decrease virulence	• No long-term protection
Targeting iron metabolism	(Alteri et al., 2009; Brumbaugh et al., 2013)	• Effective immunologic reaction against specific molecules	• Protection of the bladder and the kidneys • Reduce UTI recurrence	• Cannot target all UPEC strains (heterogeneity of the targets)
**Small compounds**				
Pillicide	(Åberg and Almqvist, 2007; Greene et al., 2014; Pinkner et al., 2006; Svensson et al., 2001)	• Prevent the formation of pili • Decrease the expression of genes related to fimbriae	• Reduce adhesion, virulence and biofilm formation of UPEC	• No *in vivo* experiments
Mannoside	(Cusumano et al., 2011; Klein et al., 2010)	• Diminution of bladder colonization • Orally bioavailable	• Reduction of the adhesion	• Clinical study in progress
Hydroxamic acid	(Griffith et al., 1978, 1988, 1991; Munakata et al., 1980; Bailie et al., 1986; Benini et al., 2000; Amtul et al., 2002; Xu et al., 2017)	• Prevent urine alkalization	• Prevent the formation of urinary stones • Decrease bladder inflammation	• Side effects (mutagenic power)
Phenyl phosphoramidates	(Texier-Maugein et al., 1987; Faraci et al., 1995; Morris and Stickler, 1998; Pope et al., 1998)	• Prevent urine alkalization	• Prevent the formation of urinary stones • Decrease bladder inflammation	• Poor stability
Capsule inhibitor	(Roberts, 1995, 1996; Llobet et al., 2008; Varki, 2008; Anderson et al., 2010; Goller et al., 2014)	• Reduce biofilm formation	• Affects a large proportion of UPEC strains	• Antigenicity in human • Poor bioavailability • Conflicting results
**Nutraceutical**				
Cranberry	(Ahuja et al., 1998; Howell et al., 2005; Freitas et al., 2006; Liu et al., 2006, 2019; AFSSA, 2007; Jepson and Craig, 2008; Pereira et al., 2011; Ermel et al., 2012; Jepson et al., 2012; Stapleton et al., 2012; Chan et al., 2013; Boonsai et al., 2014; Olczyk et al., 2014; Ulrey et al., 2014; Rafsanjany et al., 2015; Rodríguez-Pérez et al., 2016; Wojnicz et al., 2016; Pasupuleti et al., 2017; Ranfaing et al., 2018a, b; Anger et al., 2019; Bruyère et al., 2019)	• Reduction of adhesion, motility, and biofilm formation	• Impacts UPEC strains and also *P. aeruginosa*, *P. mirabilis* and *E. faecalis* • Could be used in prophylaxis	• Conflicting results
Hyaluronic acid	(Constantinides et al., 2004; Birder and de Groat, 2007; Cicione et al., 2014; Ciani et al., 2016; Torella et al., 2016; Goddard and Janssen, 2018)	• Reduction of adhesion	• Promising results in humans	• Only retrospective studies
D-mannose	(Hills et al., 2001; Klein et al., 2010; Cusumano et al., 2011; Kranjčec et al., 2014; Domenici et al., 2016)	• Reduction of adhesion	• Fast effect after oral administration	• Conflicting results
Galabiose	(Strömberg et al., 1990; Sung et al., 2001; Larsson et al., 2003)	• Reduction of adhesion	• Diminution of kidney infections	• Not enough *in vivo* results
Chinese Medical Herb and other plants	(Banu and Kumar, 2009; Gu et al., 2011; Issac Abraham et al., 2011; Tong et al., 2011; Zhao et al., 2011; Flower et al., 2015; Meng et al., 2015; Hou and Wang, 2016; Pu et al., 2016; Sharifi-Rad et al., 2016; Mazarei et al., 2017; Jaiswal et al., 2018; Mickymaray and Al Aboody, 2019)	• Reduction of adhesion	• Reduction of UTI recurrences	• Small size studies • Little safety data
**Immunomodulant agents**				
COX-2 inhibitor	(Bleidorn et al., 2010; Hannan et al., 2014; Moore et al., 2019)	• Reduction of inflammation linked to cystitis	• Substantial reduction of UTI recurrences	• No significant results in clinical trials
Green Tea Extract	(Hoshino et al., 1999; Arakawa et al., 2004; Cooper et al., 2005a, b; Reygaert and Jusufi, 2013; Bae et al., 2015)	• Reduction of inflammation	• Reduction of UTI recurrences	• Mechanisms of action unclear • Not proved in humans
**Probiotics**				
Vaginal lactobacilli	(Isolauri et al., 2001; Galdeano and Perdigón, 2006; De Vuyst and Leroy, 2007; Cribby et al., 2008; Cadieux et al., 2009; Riaz et al., 2010; Hardy et al., 2013; Kemgang et al., 2014; Di Cerbo et al., 2016; Chikindas et al., 2018; Ng et al., 2018; Koradia et al., 2019)	• Competition, reduction of adhesion and virulence	• Natural production of antimicrobial compounds • No known side effects	• Not enough *in vivo* results
*E. coli* 83972	(Hull et al., 2000; Darouiche et al., 2001, 2005; Dashiff et al., 2011; Roos et al., 2006; Trautner et al., 2007; Prasad et al., 2009; Sundén et al., 2010)	• Colonization of the bladder by avirulent strain	• Reduction of UPEC colonization	• Not enough inclusions in clinical studies
Predatory bacteria	(Stolp and Starr, 1963; Kadouri and O’Toole, 2005; Sockett, 2009; Dashiff et al., 2011; Shatzkes et al., 2015; Gupta et al., 2016)	• Decrease of bacterial number and biofilm formation	• Efficient against Gram-negative bacteria	• Not yet tested to treat UTIs
**Bacteriophages**	(Dufour et al., 2016; Sybesma et al., 2016; Leitner et al., 2017; Ferry et al., 2018; Ujmajuridze et al., 2018; Jault et al., 2019; Kuipers et al., 2019)	• Direct bacteria killing	• Interesting animal models and human case reports.	• More human studies are required

## Vaccines

Vaccines have been studied to prevent recurrent UTI in the aim not to kill infectious pathogens but to protect the host against infection by priming the immune response to uropathogens. Different vaccine strategies have considered the use of both surface antigen or inactivated whole bacterium, from uropathogens, to generate protective antibodies as a preventive strategy for recurrent UTIs. An ideal vaccine will target factors critical for establishment of bladder colonization (O’Brien et al., 2016). In this way, vaccines containing O antigens (important virulence factors that are targets of both the innate and adaptive immune systems), fimbrial subunits (responsible for the attachment to host cells, the first step of UTI), α-hemolysin (a membrane-active protein exotoxin leading to serious tissue damage), siderophores and a variety of outer membrane siderophore receptors (allowing the sequestration of iron, the main source of bacterial growth) have been developed.

### Current Vaccine Solutions

There are currently four available vaccines with established results from randomized control trials (RCT): Uro−Vaxom^®^, Urovac^®^, ExPEC4V and Uromune^®^ (Aziminia et al., 2019).

– Uro−Vaxom^®^, also known as OM−89, is comprised of bacterial extracts from 18 UPEC strains that mediated its effect by the ability of bacterial component pathogen-associated molecular patterns to non-specifically stimulate cells of the innate immune systems (Huber et al., 2000). This effect was shown in mouse models inducing an immunological defense response within the bladder. However, its use in human trials has shown conflicting results. Four placebo controlled studies (Tammen, 1990; Schulman et al., 1993; Magasi et al., 1994; Bauer et al., 2005) showed that taking one Uro-Vaxom^®^ tablet daily for 3 months significantly reduced the number of UTIs in the treatment group, with a RR = 0.61 (95%CI 0.48–0.78) of developing a UTI in the treatment group during an observation ranging from 3 to 12 months. However, a recent multicenter double-blind control trial of 451 patients showed no significant difference in UTI rates between Uro-Vaxom^®^ and the placebo (Wagenlehner et al., 2015).
– Urovac^®^ is a mucosal vaccine in the form of a vaginal suppository containing 10 different strains of heat-inactivated uropathogenic bacteria (six serotypes of *E. coli* strains, *P. vulgaris*, *Morganella morganii*, *E. faecalis* and *K. pneumoniae*) (Yang and Foley, 2019). The aim of this vaccine preparation was to incorporate a broader range of commonly implicated uropathogens and thus provide broad protection. Overall, Urovac^®^ has been shown to reduce the risk of recurrence (RR 0.75, 95% CI 0.63–0.89) (Uehling et al., 1997, 2003; Hopkins et al., 2007). This effect was more pronounced in the group that received a follow-up booster vaccination (Aziminia et al., 2019).
– ExPEC4V is composed of O−antigens of four *E. coli* serotypes (O1A, O2, O6A, and O25B) delivered as a single intra-muscular injection. These serotypes are a key immune evasion strategy used by the bacterium. This vaccine has shown good safety and immunogenicity in several phase 1 and 2 trials (Hopkins et al., 2007; Huttner et al., 2017; Huttner and Gambillara, 2018; Frenck et al., 2019).
– Uromune^®^ is a new sublingual vaccine composed of inactivated *E. coli*, *K. pneumoniae*, *Proteus vulgaris* and *E. faecalis*. This vaccine has been evaluated in three large retrospective Spanish studies and showed a 70–90% reduction of recurrence when compared to antibiotic prophylaxis (Lorenzo-Gómez et al., 2013, 2015). A prospective observational study showed that 59 out of 75 women (78%) which received 3 months of Uromune^®^ treatment as a sub-lingual spray once a day, had no new UTIs during both the treatment and the one year of follow-up period (Yang and Foley, 2018). Another recent lager prospective study showed that 65% of 784 women had 0 or 1 UTI after 6 months of daily sub-lingual administration of Uromune^®^ (Ramirez Sevilla et al., 2019).

This interesting result should be confirmed in a larger study with a control group. It has to be noted that the need for a 3-month daily administration raises questions on the immunogenicity and the expected compliance to this treatment. One international multicenter phase III RCT is currently underway.

A recent meta-analysis noted that the use of vaccines appeared to reduce UTI recurrence compared to placebo. However, the heterogeneity amongst studies renders interpretation and recommendation for routine clinical use difficult at present (Prattley et al., 2020). Further randomized controlled trials are warranted to assess the efficiency and the safety of the existing vaccines against UTIs.

### New Candidates

Several bacterial virulence factors involved in UTI are promising vaccine targets.

#### Vaccines Targeting Adhesion

Bacterial adhesion to urothelium represents a crucial step in the pathogenesis of UTIs. It permits bacteria to resist mechanical elimination by the flow of urine and bladder and increases persistence of *E. coli*. One large family of adhesive organelles are pili assembled by the chaperone-usher pathway (CUP) pili. These pili are critical virulence factors in a wide range of pathogenic bacteria, including *E. coli*. CUP pili mediate adhesion to host and environmental surfaces, facilitate invasion of host tissues, and promote interaction of bacteria with each other to form biofilms (Sauer et al., 2004). The most studied of the CUP pili are the Type 1, P, and S pili, present in UPEC and allowing the colonization of mucosal surfaces. They promote both irreversible bacterial attachment and invasion of uroepithelial cell membrane within the bladder. Interactions mediated by these adhesins can stimulate a number of host responses that can directly influence the outcome of a UTI (Mulvey, 2002). They are thus promising vaccine candidates because of the importance of this adhesion. The potential exists to develop antibodies that block the adhesin-host cell receptor interaction and thus decrease bacterial colonization (Wizemann et al., 1999).

FimH is a major determinant of the adhesive subunit of Type 1 fimbriae, which has high tropism for urinary tract receptors. It binds to mono-mannose. Lack of this compound on renal epithelia has suggested a limited role in pyelonephritis. It has been targeted as a good candidate for a vaccine because of its critical role in cystitis pathogenesis (Figure 1). A FimCH vaccine protected cynomolgus monkeys from cystitis (Langermann et al., 2000). Another study on mice with an intranasal and intramuscular administration also showed a protection against cystitis, but the intranasal method produced a stronger immune response (Poggio et al., 2006). In a more recent study, a recombinant protein MrpH.FimH (consisting of a combination between two adhesins, FimH from UPEC and MprH from *P. mirabilis*) was injected with a transurethral instillation and the authors demonstrated a high immune response and a protection against *E. coli* and *P. mirabilis* (Habibi et al., 2016).

By comparison the Type P pilus is more involved in pyelonephritis due to the presence of globoseries glycosphingolipids (the receptor of this pilus) in kidney. This P fimbria helps to cross the epithelial barrier to enter the bloodstream and can cause hemagglutination of erythrocytes (Riegman et al., 1988). Approximatively 1,000 of subunits form a P fimbria. Among them the major constituent is the protein subunit PapA, and minor subunits are PapD, PapE, PapF, and PapG. So subunit vaccines based on this pilus have been developed to block renal colonization. Attempts on using the major subunit PapA as a vaccine, although initially favorable in mice (O’Hanley et al., 1985), failed due to the poor generation of protective antibodies most probably due to natural variation in the PapA pilus subunit (Figure 1; De Ree and Van den Bosch, 1987). A new vaccine based on purified PapDG protein demonstrated a good efficiency to prevent colonization of kidney on cynomolgus monkeys (Roberts et al., 2004).

#### Vaccines Targeting Capsule

The main role of a capsule is to cover and protect *E. coli* from the host immune system. It provides protection against engulfment and complement-mediated bactericidal effect in the host (Johnson, 1991). Based on this implication in virulence strategy, capsules represent a promising vaccine target. In this way, conjugate UTI vaccines against UPEC capsule and lipopolysaccharide (LPS) components have shown protection in animal models after same-strain challenge. In early studies, intraperitoneal, subcutaneous or bladder injection of O-antigen from *E. coli* of different serotypes (O6, O8 and K13) protected rats and rhesus monkeys from pyelonephritis (Kaijser et al., 1983; Roberts et al., 1993; Kumar et al., 2005). A considerable challenge in formulating a vaccine targeting capsule or O-antigen, the most exposed component of LPS, is the great heterogeneity of serotypes among *E. coli* isolates. Indeed, six different O serotypes account for only 75% of UPEC isolates (Stenutz et al., 2006), making the formulation of a broadly protective conjugate vaccine impractical. Furthermore, some capsule serotypes, such as K1 and K5, are thought to evade the host immune response by molecular mimicry, potentially making them poor vaccine candidates. No studies on these vaccines have been conducted in humans yet.

#### Vaccines Targeting Toxins

Several toxins secreted by UPEC play a consequential role as virulence factors in UTI. They have the ability to alter the host cell signaling cascade and modulate inflammatory responses. They also contribute to the stimulation of the host cell death and the ability to access deeper tissues. Many toxins have been reported including α-hemolysin (HlyA) or cytotoxic necrotizing factor 1 (CNF1). α-hemolysin is a pore-forming toxin, it can lyse erythrocytes, and induces the apoptosis of target host cells promoting the exfoliation of bladder epithelial cells (Johnson, 1991). CNF1 stimulates actin stress fiber formation and membrane ruffle formation resulting in the entry of *E. coli* into the cells. This protein interferes with polymorphonuclear phagocytosis and provokes apoptotic death of bladder epithelial cells (Asadi Karam et al., 2019). Following their importance in virulence, the toxins produced by UPEC have been used to develop vaccines to protect the bladder and kidney during a UTI. However, as they are secreted and thus removed from the bacteria, they do not make ideal vaccine candidates. Indeed, a purified α-hemolysin toxoid vaccine prevented renal injury, but not colonization, in mice after challenge with a hemolytic UPEC strain (Figure 1; O’Hanley et al., 1991). Of note, rather than being secreted as “naked” proteins, α-hemolysin and CNF1 are associated with outer membrane vesicles (OMVs), which bleb from the surface of Gram-negative bacteria during all stages of growth (Ellis and Kuehn, 2010). OMVs also contain adhesins, enzymes, and non-protein antigens like LPS. OMVs are intriguing vaccine candidates, and because they contain LPS and other pro-inflammatory virulence factors, they should not require adjuvants to stimulate the immune system. However, no UPEC OMV vaccines have yet been tested.

#### Vaccines Targeting Iron Metabolism

The acquisition of iron is crucial for bacteria life and *E. coli* uses iron for transporting and storing oxygen, DNA synthesis, electron transport and metabolism peroxides. However, the amount of iron availability is reduced in host. In response, *E. coli* produces siderophores, molecules that mediate iron uptake. Four siderophore systems have been identified such as yersiniabatin, aerobactin, enterobacterin, and salmochelin (Johnson, 1991).

Some studies have targeted siderophore, heme receptors and other functional molecules involved in iron acquisition. In one study, the authors tested six iron receptors and found that the antibodies against the yersiniabactin receptor, FyuA, conferred kidney protection in mice (Brumbaugh et al., 2013). In another mouse model, instead of targeting iron receptors, the same authors targeted molecules involved in iron metabolism. Of the six candidates, two conferred a protection to the bladder (IreA and IutA) and one protected the kidneys (Hma) after an intranasal immunization (Alteri et al., 2009).

## Small Compounds

In the current setting small compounds are low molecular weight molecules that are typically bacterial substrates or products or mimics thereof. They can act as inhibitors by binding active sites and substrate binding sites of proteins involved in pathogenicity and so impact bacterial infections.

### Small Compounds Targeting Adhesion

As previously noted, one of the critical mechanisms for the pathogenesis of the uropathogenic bacteria is its adhesion to uroepithelium (Beerepoot and Geerlings, 2016), due to fimbriae (specially the Type 1 and the P-fimbriae), playing a role in both cystitis and pyelonephritis (Beerepoot and Geerlings, 2016; Muenzner et al., 2016). The very conserved structure of the adhesive organelles makes them good candidates to develop antibacterial agents (Piatek et al., 2013). The small molecules targeting adhesion can be classified into two categories: those inhibiting the capacity of adhesion of the fimbriae, and those targeting fimbriae assembly.

#### Pilicide

The main action of these molecules is to prevent the formation of UPEC pili by decreasing the levels of Type 1 and P piliation (Åberg and Almqvist, 2007). Pilicides are small molecules which have a ring-fused 2-pyridone backbone. Some pilicides act directly on pili assembly chaperones, through adhering to their hydrophobic substrate binding sites (Svensson et al., 2001; Pinkner et al., 2006). Others interfere with the transcription of pili genes and some cases genes involved in flagella biogenesis such as the pilicide ec240, the most potent inhibitor of Type 1 piliation and of type 1 pilus-dependent biofilm formation to date (Greene et al., 2014).

*In vitro* studies testing the potential of pilicides have shown promising results. These compounds decreased (i) the adhesion of UPEC strains on cells by a strong reduction (70–80%) of fimbriae density (Piatek et al., 2013), and (ii) the ability to form Type 1 pilus dependent biofilms (Pinkner et al., 2006). In a mouse study, the pilicide had a strong impact on adhesion and biofilm formation and also reduced the virulence *in vivo*. It is also interesting to note that it reduced the biofilm formation on abiotic surface (Cegelski et al., 2010).

To develop this compound into a therapeutic, further studies are needed to assess its pharmacokinetics and pharmacodynamics and to determine the concentration at which it accumulates in the bladder or other potential sites of infection.

### Small Compounds Targeting Urease

Urease, an enzyme which catalyzes the hydrolysis of urea, is crucial in the pathogenesis of several uropathogenic bacteria such as *P. mirabilis*, *Klebsiella* sp., *Pseudomonas* sp. and *Staphylococcus* sp. (Mobley and Hausinger, 1989). This enzyme leads to the alkalinization of the urine and the production of struvite and carbonate apatite that make up the major component of urinary stones (Burne and Chen, 2000). These conditions lead to the inflammation of the urogenital epithelia thus increasing the risk of catheter-associated biofilm formation that may contribute to pyelonephritis (Musher et al., 1975; Jacobsen et al., 2008), mainly due to both bacterial and host cysteine protease (Xu et al., 2017).

The most studied inhibitors of urease are hydroxamic acids (Amtul et al., 2002; Figure 1). These molecules have a high inhibitory activity against urease, by bonding to the two nickel ions in the urease active site (Benini et al., 2000). Initially, these molecules were used to treat UTIs by preventing urine alkalization (Griffith et al., 1978, 1988). However, because of the growing evidence of side effects such as mutagenic power, they were progressively phased out (Munakata et al., 1980; Bailie et al., 1986; Griffith et al., 1991).

Through similarly interacting with nickel ions in the urease active site, the phenyl phosphoramidates were found to have the highest inhibitory activity (Faraci et al., 1995). Studies testing these molecules in an *in vitro* model (Morris and Stickler, 1998) and in a rat model (Texier-Maugein et al., 1987) found promising results. Since then, no *in vivo* studies or clinical trials have been developed, probably due to the poor hydrolytic stability of these molecules which leads to a very short half-life (Pope et al., 1998).

Other molecules that possess inhibitory activity against bacterial urease have been developed, but they are not fully adapted to treat UTIs. One of them is the quinones, a class of active compounds with a high oxidizing potency (Zaborska et al., 2002). These molecules had not been evaluated *in vivo* models due to their cytotoxic and cancerogenic properties.

### Small Compounds Targeting Bacterial Capsule

Polysaccharide capsule biogenesis plays an important role in UPEC virulence. Like other pathogens, the capsule is used as a defense against opsonophagocytosis and complement-mediated killing (Roberts, 1995, 1996). The capsule is also involved in the formation of biofilm and the formation of intracellular bacterial communities (Llobet et al., 2008; Anderson et al., 2010). Until now, the human use of small-molecule inhibitors of UPEC capsule biogenesis was not available because of their antigenicity and poor bioavailability (Varki, 2008). Nevertheless, mouse studies identified two active agents (DU003 and DU01), that caused significant bacterial death (Goller et al., 2014; Figure 1).

## Nutraceuticals

Nutraceuticals are pharmaceutical alternatives, consisting of all the foods or food products which provide medical benefits and can be delivered under medical form. They provide health benefits in addition to their basic nutritional value.

### Cranberry (*Vaccinium macrocarpon*)

Cranberry (*Vaccinium macrocarpon* Ait.) is a berry that grows in North America. In recent years, the use of cranberry has increased in the prophylactic approach of recurrent UTI (Howell et al., 2005). Although, its mechanism of action is unclear, there are several possible targets of cranberry (Figure 1).

The main efficacy is related to the antiadherence properties of cranberry (Ahuja et al., 1998; Liu et al., 2006) due to the A-type proanthocyanidin (PAC-A) that has been shown to be an important inhibitor of Type-I fimbriae *E. coli* adhesion to uroepithelial cells. Some *in vitro* and *in vivo* studies demonstrated the capacity of the cranberry to reduce the adhesion of bacteria to the cells (Ermel et al., 2012; Rafsanjany et al., 2015; Liu et al., 2019). Cranberry has also shown convincing results on motility and biofilm formation. Indeed, it has a negative impact on the swarming of *Pseudomonas aeruginosa* and *P. mirabilis* (Chan et al., 2013) and on the biofilm formation of *E. faecalis*, *P. aeruginosa* and *E. coli* (Ulrey et al., 2014; Rodríguez-Pérez et al., 2016; Wojnicz et al., 2016).

The use of cranberry has been associated with a decrease in the incidence of UTIs, although some conflicting results have been reported in the literature. Although cranberry products have been shown to significantly reduce the incidence of UTIs at 12 months (RR 0.65, 95% CI 0.46–0.90) compared with placebo/control in women with recurrent UTIs in a Cochrane review from 2008 (Jepson and Craig, 2008), an updated review concluded that cranberry products did not show any significant reduction in the occurrence of symptomatic UTI in the same population (Jepson et al., 2012).

A placebo controlled trial, published after the last review, showed that women randomized to cranberry juice had a non-significant reduction in numbers of P-fimbriated *E. coli* in urine and in the rate of symptomatic UTIs (Stapleton et al., 2012).

Because dosage, concentration and formulation of PAC-A are not well defined, the conflicting results may be explained by the difference in PAC-A concentration between cranberry formulations (juice, beverage, tablets) in the different studies making it difficult to choose one formulation over another (Anger et al., 2019).

It must be noted that many of the products containing cranberry used in studies are only for research purposes, limiting the prophylactic application of cranberries beyond research (Stapleton et al., 2012).

Finally, a recent publication showed that the cranberry proanthocyanidins had a variable effect on a collection of *E. coli* strains that could explain the discordant results observed in the clinical studies (Ranfaing et al., 2018a).

In order to enhance the effectiveness of cranberry, combinations of cranberry and other natural products with antimicrobial properties could be used, such as propolis. Propolis is a resinous material collected by bees from plants then mixed with wax and bee enzymes (AFSSA, 2007). Propolis has antimicrobial, anti-inflammatory, anti-tumoral, immune-modulatory and anti-oxidant activities (Boonsai et al., 2014). It has been used for several years to treat gastrointestinal disorders (food supplement) (Freitas et al., 2006), to promote oral health (mouthwash) (Pereira et al., 2011) and in dermatological care (creams and ointments) (Olczyk et al., 2014; Pasupuleti et al., 2017; Ranfaing et al., 2018b). *In vitro* studies showed that propolis potentiated the effect of cranberry proanthocyanidins on adhesion, motility (swarming and swimming), biofilm formation (early formation and fully-formed biofilm), iron metabolism and stress response of UPEC (Olczyk et al., 2014; Pasupuleti et al., 2017; Ranfaing et al., 2018a, b). Moreover, this association was active in all the *E. coli* strains studied, ruling out the variable effect observed with the cranberry used alone (Ranfaing et al., 2018a).

A recent RCT versus placebo in 85 women with recurrent UTIs showed a slight reduction in the number of cystitis events in the first 3 months in the propolis and cranberry group after adjustment on water consumption (0.7 vs. 1.3, *p* = 0.02), but no difference in the mean number of infections in women with at least one infection. Of note, the mean time to onset of the first cystitis episode was significantly longer in the propolis + cranberry group (70 vs. 43 days, *p* = 0.03) and tolerance to the treatment was similar in both groups (Bruyère et al., 2019).

### Hyaluronic Acid

The urinary bladder epithelium is composed of urothelial cells which carry specific sensors and properties as well as forming the first barrier to pathogens (Birder and de Groat, 2007). To maintain this capacity to fight infections, these cells produce sulfated polysaccharide glycosaminoglycan (GAG) which covers the epithelium and forms a non-specific anti-adherence factor. A major proportion of the GAG layer of the bladder is composed of hyaluronic acid (HA) and chondroitin sulfate (CS). Virulence factors (secreted by *E. coli* for example) damage the GAG layer to prepare its adhesion (Constantinides et al., 2004; Figure 1). One strategy for the management of UTI is based on the re-establishment of the GAG layer of the bladder epithelium with intravesical instillations of HA alone or in combination with CS. Various randomized and non-randomized studies have been performed.

A study investigated the impact of HA and CS on recurrent UTIs on 276 women (aged 18–75 years). The intravesical administration of HA and CS was given to 181 women and the standard treatment against recurrent UTIs was given to 95 women. There was a 49% reduction in the rate of recurrence (defined as one bacteriologically confirmed UTI in the year following the treatment initiation) in patients treated with HA + CS compared with standard care [adjusted OR 0.51 (95%CI 0.27–0.96)]. However, no significant difference was found when considering the number of recurrences or the median time to first recurrence (Ciani et al., 2016).

Another retrospective study in 157 women found similar results with a significant reduction in UTI recurrence and an increased time-to-recurrence between UTIs (Cicione et al., 2014).

It has to be noted that the administration protocol (different between participating centers) of weekly instillation for one month followed by monthly instillations may be a limiting factor for patients.

A synergy might exist between HA + CS and estrogen. This association has been explored in 145 postmenopausal women with mild-to-moderate urogenital atrophy and a history of recurrent UTI. Participants were divided into three groups: vaginal estrogen, oral HA, and oral HA + CS and vaginal estrogen. Oral treatments were effective in preventing recurrent UTI (number of patients with fewer than two infective episodes in the 6-month follow-up and fewer than three episodes in the 12-month follow-up), especially if administered with vaginal estrogen therapy. A slight effect on the HA alone but also a significant impact of the estrogen and the HA + CS on the recurrence of UTIs in postmenopausal women was observed (Torella et al., 2016).

Recently a meta-analysis suggested that HA ± CS decreased the rate of UTI recurrence and increased the time to recurrence (Goddard and Janssen, 2018). Moreover the authors noted the safety of HA therapy even if the intravesical instillation is more invasive than other administrations (usually *per os*). The combination therapy was more performant than the use of HA alone. It seems essential to perform a well-designed, randomized, controlled clinical trials with larger population.

### D-mannose

As seen above, CUP pili are important virulence factors and represent optimal targets for antivirulence compound development. *E. coli* binds to mannosylated host cells via their mannose-binding lectin domains of FimH.

Two developments have been proposed to prevent this interaction:

– D-mannose is a monosaccharide closely related to glucose. It blocks the adhesion by high affinity binding to the FimH, thus preventing FimH from binding host mannose on urinary tract surfaces. Absorption after oral administration is fast (30 min to reach the organs) and it is eliminated via the urinary tract (Hills et al., 2001).
– The structure of the FimH adhesin bound to mannosilable proteins has been used to design mannosides, These molecules block FimH function by binding in the FimH mannose-binding pocket (Pinkner et al., 2006; Domenici et al., 2016). Mannosides are also potent inhibitors of biofilm formation *in vitro*. Some mouse models demonstrated a decrease in bladder colonization after an oral administration of mannosides and a prevention of acute and chronic UTI (Klein et al., 2010; Cusumano et al., 2011; Figure 1). In this way, an exogenous intake of D-mannose competitively blocks the interaction between the bacterial fimbriae and host cells (Cusumano et al., 2011; Figure 1). One RCT compared the recurrence of UTIs in a group of patients taking daily nitrofurantoin to a group treated with daily D-mannose powder. The risk of recurrence was similar between groups, whilst there was a reduction of the side effects in the D-mannose group (Kranjčec et al., 2014).

### Galabiose

Type P fimbriae adhere to the galabiose-like receptor via PapG (Larsson et al., 2003). As previously noted, this kind of fimbriae is essential for the pathogenesis of UTIs in helping the bacteria to reach the kidneys (Strömberg et al., 1990). Better understanding of the structure of the different variants of PapG (mostly PapG II and PapG III) might lead to drugs designed to target this adhesin (Sung et al., 2001; Figure 1). No *in vivo* studies on the impact of this sugar on UTIs have yet been performed. Evaluation on the most prevalent uropathogenic bacteria is essential to clearly evaluate the potential of galabiose to reduce UTI.

### Vitamin C

Vitamin C (ascorbic acid) is known to possess antioxidant and antimicrobial activities. As microbial infections cause reactive oxygen species (ROS) release by phagocytes, it is helpful in the limitation of infection through deactivation of microorganism killing. ROS may also cause damage to the host cells, therefore the level of ROS released by phagocytes should be reduced directly after infection (Liu et al., 2018). Vitamin C is an essential co-enzyme in the oxidative stress pathways, capable of ROS removal. Habash et al. (1999) suggested that vitamin C decreased the adhesion and microorganisms colonization of the biomaterials used in diagnostic/treatment procedures involving the urinary tract.

Moreover, two trials have studied the use of vitamin C to prevent UTIs. In a single-blind randomized study in thirteen spinal cord injury patients randomized to placebo or 500 mg ascorbic acid four times daily, there was no clinical benefit on urinary infection from the use of ascorbic acid (Castelló et al., 1996). In a second single-blind randomized trial in 110 pregnant women, participants taking a vitamin regimen with 100 mg ascorbic acid per day for 3 months showed a reduction in symptomatic UTIs incidence from 29.1 to 12.7% compared to participants following a vitamin regimen without ascorbic acid (Ochoa-Brust et al., 2007).

To date, there is no evidence of vitamin C action in the prevention of UTI.

### Chinese Herbal Medicine (CHM)

Chinese Herbal Medicine (CHM) is the ancient art of compiling complex herbal formulae usually comprising up to 15 herbs. CHM has been historically used to treat UTI. Some frequently used Chinese herbs are known to have significant diuretic, antibiotic, immune enhancing, antipyretic, anti-inflammatory and pain relieving activities. Some of these herbs have shown an *in vitro* inhibitory activity against several uropathogens, especially against *E. coli* in decreasing its adherence to bladder epithelial cells (Tong et al., 2011). In an antibacterial test against mice, it was found that Sanjin tablets (composed by five kinds of CHM) have strong bacteriostasis activity (Hou and Wang, 2016). This product is used to treat acute uncomplicated lower UTI (Meng et al., 2015) and to reduce the symptoms of chronic UTI by reducing the secretory level of some urinary cytokines (Hou and Wang, 2016). A first meta-analysis of three RCTs including 282 women suggested that CHM significantly reduced recurrent UTI rates compared to antibiotics (RR 0.28, 95%CI [0.09 to 0.82]) (Flower et al., 2015). The second one has concluded that the current evidence is insufficient to support the efficacity and safety of Sanjin tablets for acute uncomplicated lower UTI (Pu et al., 2016). Only two of these RCTs reported adverse events (Gu et al., 2011; Zhao et al., 2011). Neither found any liver or renal impairment. Further studies are needed to definitively evaluate the potential of CHM on prevention/treatment of UTI.

#### Other Phytochemicals

For centuries, plants have been used as alternative and traditional medicine around the world notably as therapies for infectious diseases (Banu and Kumar, 2009; Sharifi-Rad et al., 2016). Plants and their secondary metabolic derivatives are a major resource of antioxidants due to the presence of phenolic compounds including flavonoids, phenolic acids, or tannins. Different extracts from plants and spices have demonstrated anti-inflammatory, antimicrobial and diuretic activities (Mickymaray and Al Aboody, 2019). They also exhibited anti-quorum sensing and anti-biofilm potentials (Issac Abraham et al., 2011; Mazarei et al., 2017). One of the main mechanism of action is the antiadhesive action due to the formation of H-bonds between the FimH protein ligand and the plant compounds (Jaiswal et al., 2018). Future clinical trials must be done after complete pharmacokinetic/pharmacodynamic analyses.

## Immunomodulant Agents

The innate immune system activation through the secretion of cytokines and the recruitment of macrophages and neutrophils rapidly occurs after the onset of UTI (Duell et al., 2012). Even in the absence of an effective antibiotic treatment, this immune reaction might be enough to counter the infection. In the case of the persistence of several bacterial strains in the bladder, persistent acute immune response and tissue inflammation can be observed. Moreover, multiple infections can lead to chronic inflammation that increases the risk of developing recurrent UTIs (Ferry et al., 2004; Hannan et al., 2010). There is an increase in the expression of cyclooxygenase (COX)-2 after an infection of the bladder epithelial cells by UPEC. Moreover, there is a correlation between the severity of the inflammation and the increase of COX-2 expression (Chen et al., 2011; Figure 1). COX-2 inhibition prevents urothelial transmigration by neutrophils and damage to the urothelial barrier and facilitates the innate responses (Hannan et al., 2014). A double-blind RCT on 79 women with uncomplicated UTI showed equivalent symptoms at Day 4, when taking ibuprofen (200 mg t.i.d) compared to ciprofloxacin (Bleidorn et al., 2010).

A recent 2 × 2 factorial placebo RCT evaluating ibuprofen in 382 women demonstrated a substantial reduction in antibiotic use in patients taking ibuprofen without differences in terms of symptom relief or speed of recovery (Moore et al., 2019).

Plant-based immunomodulants such as Green Tea Extract (GTE) have also shown promise (Bae et al., 2015). GTE contains an array of polyphenolic compounds, especially catechins. The biological properties of catechins are antioxidant, antiangiogenesis, antiproliferative activity, and antineoplastic (Cooper et al., 2005a, b). Some *in vitro* studies showed an antibacterial impact of GTE against UPEC (Hoshino et al., 1999; Arakawa et al., 2004; Reygaert and Jusufi, 2013) and in a rat model of cystitis, catechins significantly decreased inflammation and uroepithelium edema (Noormandi and Dabaghzadeh, 2015). These results are promising even though the mechanisms of action are still unclear.

## Probiotics

A probiotic is a live microorganism that confers a health benefit.

### Vaginal Lactobacilli

*Lactobacilli* are frequently dominant microorganisms in vaginal flora (Mendling, 2016). Different observations can be done: (i) they have the ability to interfere with the adherence, growth and colonization of UPEC (Falagas et al., 2006); (ii) a change in the normal vaginal flora has been shown to facilitate the recurrence of UTIs (Cribby et al., 2008); and (iii) the use of commensal bacteria (such as *Lactobacilli*) reduces the proportion of uropathogens and thus restores bacterial homeostasis (Hardy et al., 2013). Although their exact mechanisms of action are still unknown, lactobacilli strains seem to have at least three different modes of action (Ng et al., 2018; Figure 1):

– The first one is the bacteriostatic effect due to the direct competition of probiotics with uropathogens in terms of nutrient and attachment sites (Di Cerbo et al., 2016).
– The second is the impact of probiotics on uropathogens virulence through the ability of *Lactobacillus* byproducts (such as lactic acid and hydrogen peroxide) to downregulate the expression of virulence genes. This has been illustrated in an *in vitro* study in which *Lactobacillus* byproducts inhibited the expression of Type 1- and P-fimbriae-encoding genes in *E. coli*, disrupting adhesion and invasion capacity (Cadieux et al., 2009).
– The third is the bactericidal effect of *Lactobacillus* on uropathogens. This effect can be achieved through the production of antimicrobial peptides known as bacteriocins. These bacteriocins reduce the number of uropathogens (Chikindas et al., 2018) in a strain-specific manner (De Vuyst and Leroy, 2007). *Lactobacillus* species that produce bacteriocins against *E. coli* have been identified (Riaz et al., 2010).

Probiotics can also modulate the immune system. In addition, bacterial strains secreting “immunomodulins” and cytokines are able to reduce infection by pathogenic bacteria (Galdeano and Perdigón, 2006; Kemgang et al., 2014). *Lactobacillus* species have these anti-inflammatory and immune-regulatory actions (Isolauri et al., 2001).

A non-inferiority randomized trial compared antibiotic prophylaxis and *Lactobacillus* prophylaxis in 252 postmenopausal women with recurrent UTIs who received 12 months of prophylaxis with trimethoprim-sulfamethoxazole (TMP-SMX), 480 mg once daily or oral capsules containing 10^9^ CFU of *Lactobacillus rhamnosus* GR-1 and *Lactobacillus reuteri* RC-14 twice daily (Beerepoot et al., 2012b). The *Lactobacillus* treatment did not demonstrate non- inferiority, with an average number of symptomatic UTIs during the year of follow-up of 2.9 in the antibiotic group versus 3.3 in the lactobacilli group. The benefit of Lactobacilli was that it had no impact on antibiotic resistance compared to trimethoprim- sulfamethoxazole.

A reduction in the recurrence rate of UTI with the use of lactobacilli products (pooled RR = 0.68, 95%CI 0.44 to 0.93, *p* < 0.001) (Ng et al., 2018) was underlined in a meta-analysis including 620 patients. In this study, two intravaginal suppositories (containing *Lactobacillus crispatus* CTV05, *Lactobacillus rhamnosus* GR1 and *Lactobacillus reuteri* RC14) had the highest efficacy (Ng et al., 2018).

A recent randomized, double-blind, placebo-controlled pilot study in 81 premenopausal women showed that the administration of Bio-Kult Pro-Cyan (a commercially available product containing probiotic strains (*Lactobacillus acidophilus* PXN 35, *Lactobacillus plantarum* PXN 47) and cranberry extract (36 mg/d PACs) twice-daily for 26 weeks, led to significantly lower number of recurrent UTIs compared to placebo (9.1 vs. 33.3%; *P* = 0.0053) (Koradia et al., 2019).

### Bacterial Interference: *Escherichia coli* Strain 83972

The intentional colonization of the bladder with a non-virulent strain, also called bacterial interference, has been studied among patients with neurogenic bladder. *E. coli* 83972 is a clinical strain, isolated from a woman with chronic urinary colonization and which has naturally lost its capacity to develop Type 1 and Type P fimbriae. This strain has been used for prophylactic purposes to deliberately colonized the bladders with this bacterium to prevent colonization/infection by pathogenic species.

In a mouse model of UTI, *E. coli* 83972 demonstrated a better fitness than a virulent strain of UPEC. In a poor environment, like the bladder, this difference in fitness is a crucial advantage for the competition between bacteria. The 83972 strain could reduce the impact of UTIs by a monopolization of resources and space (Roos et al., 2006).

Seven clinical studies are available: three are RCT, one of which is a crossover designed study; and four are prospective cohorts (Hull et al., 2000; Darouiche et al., 2001, 2005; Dashiff et al., 2011; Trautner et al., 2007; Prasad et al., 2009; Sundén et al., 2010). Sample sizes were small and varied from 12 to 44 patients. Clinical endpoints were the interval before first recurrence or the incidence of UTI during follow up.

Despite this heterogeneity, all studies demonstrated the ability of non-virulent strain to protect patients from UTI. One limit is the difficulty to achieve bladder colonization with the non-virulent strain (only 38% of patients in one of the RCT) (Darouiche et al., 2011).

### Predatory Bacteria

Predatory bacteria are small, motile, deltaproteobacteria that are a predatory invader of other Gram-negative bacteria (Figure 1). They occupy an intraperiplasmic niche and kill, digest and lyse their host, the prey cell. *Bdellovibrio* and *Micavibrio* are the most studied predatory bacteria (Stolp and Starr, 1963). *Bdellovibrio bacteriovorus* uses its type IV pilus to adhere and penetrate the outer membrane (bdelopast) of its prey. Inside the bacteria, *B. bacteriovorus* modifies the membrane of its prey to allow its growth until it uses all the nutrients. This entire process takes only 2–3 h (Sockett, 2009). Several Gram-negative human pathogenic bacteria such as *E. coli*, *Klebsiella* spp. and *Pseudomonas* spp. can be targeted by these predatory bacteria (Dashiff et al., 2011). Furthermore, it has been showed *in vitro* that *B. bacteriovorus* significantly reduced the quantity of biofilm (Kadouri and O’Toole, 2005).

*In vitro* and mouse model studies have underlined the fact that the predatory bacteria have no negative impact on human cell lines (Gupta et al., 2016) nor on animals (Shatzkes et al., 2015). *B. dellovibrioa* is thus a potential therapeutic agent, but has not been yet applied in this way. No studies have investigated *B. bacteriovorus* to treat UTIs to date. However, these bacteria offer an exciting path for further research where *in vivo* studies should be the focus.

## Bacteriophages

Bacteriophages are viruses that parasitize a bacterium by infecting it and reproducing inside it and can act as bactericidal agents. Bacteriophage therapy is currently applied in different areas of medical research. Indeed, it has been recognized as an alternative treatment in localized infections such as otitis, infected burns and osteoarticular infections (Ferry et al., 2018; Jault et al., 2019). However, its use in UTI is scarce.

One research team has studied the combination of transurethral resection of prostate with bacteriophage therapy used instead of per operative antibiotics. They first demonstrated the *in vitro* lytic activity of commercial bacteriophage cocktails on 41 *E. coli* and 9 *K. pneumoniae* strains. The lytic activity of the bacteriophage cocktails varied between 66% and 93%. They also showed the potential of bacteriophage adaptation experiments to increase the lytic activity, leading to an increase from 66 to 93% for one of the cocktails (Sybesma et al., 2016).

In an animal model, Dufour et al. (2016) showed the ability of phage to treat an *E. coli* UTI. The administration of the same bacteriophage cocktail showed a significant reduction of the bacterial load in the *E. coli* kidney infection model as well as in the *E. coli* pneumonia model, but not in an *E. coli* sepsis model.

*In vivo* studies were performed with a commercial preparation called Pyo bacteriophage, composed of bacteriophage lines active against a broad spectrum of uropathogenic bacteria: *S. aureus*, *E. coli*, *Streptococcus* spp. (including *Enterococcus* spp.), *P. aeruginosa*, and *Proteus spp.* in nine patients planned for transurethral resection. Bacteria load decreased in two-thirds of the patients (6/9), without any associated adverse events (Ujmajuridze et al., 2018).

One case report described the treatment of a recurrent *P. aeruginosa* UTI associated with a bilateral ureteral stent. A phage cocktail containing phages with activity against *Streptococcus pyogenes*, *S aureus*, *E. coli*, *P. aeruginosa*, *P. vulgaris*, and *P. mirabilis* was administered. After 6 days of treatment, meropenem and colistin were started for 30 days. The results showed a 10-fold reduction of bacteria load in the urine after the first 5 days of phage treatment. The bacterial load was undetectable after 2 days of subsequent antibiotic treatment. Urine samples remained sterile until 1 year after the end of the antibiotic treatment.

Another case report showed the treatment of a recurrent UTI with an ESBL-producing *K. pneumoniae* in a renal transplant patient whose infection evolved into an epididymitis. The patient was definitively cured after an oral and intravesical bacteriophage treatment of 10 days following 6-week meropenem treatment (Kuipers et al., 2019).

One randomized, double-blind trial versus placebo in patients planned for transurethral resection of the prostate with UTI is ongoing. Patients will be randomized in a 1:1:1 ratio to receive 7 days of either: (i) bacteriophage (Pyo bacteriophage) solution, (ii) placebo solution, or (iii) antibiotic treatment (Leitner et al., 2017).

More human studies are warranted to further define the role of this treatment option in UTIs.

## Conclusion

Although prophylactic antibiotics remain the preferred preventive treatment in recurrent UTIs, the emergence of antimicrobial resistance worldwide has made the development of non-antibiotics strategies a priority. The better understanding of UTI mechanisms will help direct future research on the topic. Indeed, recent studies have revealed that infection with UPEC and a number of other Gram-negative uropathogens proceeds through dynamic intracellular and extracellular host niches during the course of acute and chronic infection.

Several targets such as uropathogenic adhesins, toxins, urease, iron metabolism and motility have been explored. Although non-antibiotic prophylactic agents appear to be well tolerated and do not seem to increase the antimicrobial resistance of the commensal flora, most of therapeutic options displayed in this review are still preliminary.

Other approaches have also been evaluated in prevention of CAUTI. In case of prolonged utilization of catheter, some techniques have been developed to prevent bacterial growth and biofilm formation. These techniques, including the devices with antimicrobial coatings such as silver, peptides, enzymes or bacteriophages, provide minimal reduction in infection incidence and will need further assessment (Percival et al., 2015; Al-Qahtani et al., 2019).

While *Lactobacillus*-containing products appear to be the most promising new alternative to currently used antibiotics, cranberry products combined with propolis would need further investigations. Studies should now be designed to investigate the interaction between non-antibiotic therapies, uropathogens and the host immune system. Importantly, clinical trials must use standardized definitions of UTI (infection versus colonization based on urinary symptoms), treatment regimens and control groups as well as an assessment of the risk/benefit ratio especially on tolerance and antibiotic resistance and an economic evaluation of the reviewed therapeutics versus prolonged antibiotic treatments. Likewise, further research is needed for vaccines, which have shown potential in initial trials.

## Author Contributions

PL, JR, J-PL, and AS wrote the manuscript. AD, CD-R, LB, and FB critically reviewed the manuscript. All authors contributed to the article and approved the submitted version.

## Conflict of Interest

The authors declare that the research was conducted in the absence of any commercial or financial relationships that could be construed as a potential conflict of interest.
